# Mutation and deletion analysis of GFR**α**-1, encoding the co-receptor for the GDNF/RET complex, in human brain tumours

**DOI:** 10.1038/sj.bjc.6690367

**Published:** 1999-05-01

**Authors:** O Gimm, A Gössling, D J Marsh, P L M Dahia, L M Mulligan, A von Deimling, C Eng

**Affiliations:** Translational Research Laboratory, Department of Adult Oncology, Charles A Dana Human Cancer Genetics Unit, Dana-Farber Cancer Institute, Department of Medicine, Harvard Medical School, 44 Binney St, SM822C, Boston, MA 02115 and Human Cancer Genetics Program, Ohio State University Comprehensive Cancer Center, 420 W 12th Avenue, 690C MRF, Columbus, OH 43210, USA; Institut für Neuropathologie, Sigmund-Freud-Straβe 25, Universitätskliniken Bonn, 53105 Bonn, Germany; Departments of Pathology and Paediatrics, 20 Barrie St., Queen's University, Kingston, ON K7L 3N6, Canada; Cancer Research Campaign Human Cancer Genetics Research Group, University of Cambridge, Cambridge, CB2 2QQ, UK

**Keywords:** *GDNF*, *GDNFRα*, *GFRα-1*, *RET*, brain tumours

## Abstract

Glial cell line-derived neurotrophic factor (GDNF) plays a key role in the control of vertebrate neuron survival and differentiation in both the central and peripheral nervous systems. GDNF preferentially binds to GFRα-1 which then interacts with the receptor tyrosine kinase RET. We investigated a panel of 36 independent cases of mainly advanced sporadic brain tumours for the presence of mutations in *GDNF* and *GFRα-1*. No mutations were found in the coding region of *GDNF*. We identified six previously described *GFRα-1* polymorphisms, two of which lead to an amino acid change. In 15 of 36 brain tumours, all polymorphic variants appeared to be homozygous. Of these 15 tumours, one also had a rare, apparently homozygous, sequence variant at codon 361. Because of the rarity of the combination of homozygous sequence variants, analysis for hemizygous deletion was pursued in the 15 samples and loss of heterozygosity was found in 11 tumours. Our data suggest that intragenic point mutations of *GDNF* or *GFRα-1* are not a common aetiologic event in brain tumours. However, either deletion of *GFRα-1* and/or nearby genes may contribute to the pathogenesis of these tumours. © 1999 Cancer Research Campaign


*British Journal of Cancer* (1999) **80**(3/4), 383–386
© 1999 Cancer Research Campaign
Article no. bjoc.1998.0367

### Mutation and deletion analysis of GFRα-1, encoding the co-receptor for the GDNF/RET complex, in human brain tumours

**O Gimm[1]*, A Gössling[1,2]*, DJ Marsh[1], PLM Dahia[1], LM Mulligan[3], A von Deimling[2] and C Eng[1,4]**

[1]Translational Research Laboratory, Department of Adult Oncology, Charles A Dana Human Cancer Genetics Unit, Dana-Farber Cancer Institute, Department of Medicine, Harvard Medical School, 44 Binney St, SM822C, Boston, MA 02115, and Human Cancer Genetics Program, Ohio State University Comprehensive Cancer Center, 420 W 12th Avenue, 690C MRF, Columbus, OH 43210, USA; [2]Institut für Neuropathologie, Sigmund-Freud-Straβe 25, Universitätskliniken Bonn, 53105 Bonn, Germany; [3]Departments of Pathology and Paediatrics, 20 Barrie St., Queen's University, Kingston, ON K7L 3N6, Canada; [4]Cancer Research Campaign Human Cancer Genetics Research Group, University of Cambridge, Cambridge CB2 2QQ, UK

**Summary** Glial cell line-derived neurotrophic factor (GDNF) plays a key role in the control of vertebrate neuron survival and differentiation in both the central and peripheral nervous systems. GDNF preferentially binds to GFRα-1 which then interacts with the receptor tyrosine kinase RET. We investigated a panel of 36 independent cases of mainly advanced sporadic brain tumours for the presence of mutations in *GDNF* and *GFRα-1*. No mutations were found in the coding region of *GDNF*. We identified six previously described *GFRα-1* polymorphisms, two of which lead to an amino acid change. In 15 of 36 brain tumours, all polymorphic variants appeared to be homozygous. Of these 15 tumours, one also had a rare, apparently homozygous, sequence variant at codon 361. Because of the rarity of the combination of homozygous sequence variants, analysis for hemizygous deletion was pursued in the 15 samples and loss of heterozygosity was found in 11 tumours. Our data suggest that intragenic point mutations of *GDNF* or *GFRα-1* are not a common aetiologic event in brain tumours. However, either deletion of *GFRα-1* and/or nearby genes may contribute to the pathogenesis of these tumours.

**Keywords:** *GDNF*; *GDNFRα*; *GFRα-1*; *RET*; brain tumours

Several receptor molecules, and their growth factor ligands, are expressed in the embryonic brain and are thought to play central roles in the development and differentiation of the nervous system (Weiner, 1995). In addition, altered function, or loss of function, of either receptor or ligand may lead to the development of malignancies of the nervous system (Weiner, 1995). Oncogenes (e.g. the gene encoding the epidermal growth factor receptor, *EGFR*) as well as tumour suppressor genes (e.g. *p53*, *PTEN*) are known to be involved in the development of brain tumours (Bogler et al, 1995; von Deimling et al, 1995; Wang et al, 1997; Duerr et al, 1998; Peters et al, 1998). Members of one broad class of growth factor receptors, the receptor tyrosine kinases (RTKs), have been shown to be involved in proliferation and differentiation during central nervous system (CNS) development (Weiner, 1995). The *RET* proto-oncogene encodes a RTK expressed in neural and neuroendocrine tissues (Takahashi and Cooper, 1987). To date, three related ligands for RET have been identified: glial cell line derived neurotrophic factor (GDNF), neurturin (NTN) and persephin (Durbec et al, 1996; Jing et al, 1996; Kotzbauer et al, 1996; Treanor et al, 1996; Trupp et al, 1996; Vega et al, 1996; Sanicola et al, 1997; Milbrandt et al, 1998). The receptor complex for GDNF, NTN and persephin comprises one of at least four membrane-bound adaptor molecules and RET (Jing et al, 1997; Thompson et al, 1998). GDNF preferentially binds GFRα-1 (*G*DNF *F*amily *R*eceptor alpha one, also known as GDNFR-α, RETL1 and TrnR1) with high affinity before this complex can interact with RET to effect downstream signalling (Jing et al, 1996; Treanor et al, 1996; Trupp et al, 1996; Vega et al, 1996; Davies et al, 1997; Sanicola et al, 1997). Similarly, NTN binds a membrane-bound adaptor GFRα-2 (related to GFRα-1 and also known as GDNFR-β, NTNR-α, RETL2 and TrnR2) with subsequent binding of RET (Baloh et al, 1997; Buj-Bello et al, 1997; Klein et al, 1997; Sanicola et al, 1997). GDNF can bind GFRα-2 as well, but with lower affinity, just as NTN can also bind GFRα-1 (Jing et al, 1997; Sanicola et al, 1997). A third co-receptor belonging to the same family, GFRα-3, has been identified, although formal binding studies have yet to be reported (Jing et al, 1997; Naveilhan et al, 1998; Worby et al, 1998). Recently, a fourth co-receptor, GFRα-4, was isolated that seems to be more closely related to GFRα-1 and GFRα-2 than to GFRα-3 (Thompson et al, 1998). Together with RET, GFRα-4 forms a functional receptor complex for persephin (Enokido et al, 1998).

Mice lacking RET or GDNF have been shown to have defects in the enteric nervous system and components of the peripheral nervous system (Schuchardt et al, 1994; Moore et al, 1996; Pichel et al, 1996; Sanchez et al, 1996). GDNF, like NTN, was initially isolated due to its ability to sustain the survival of embryonic dopaminergic neurons in vitro (Lin et al, 1993). In vivo studies subsequently demonstrated that GDNF was a target-derived trophic factor for dopaminergic neurons (Stromberg et al, 1993; Hudson et al, 1995; Tomac et al, 1995). In several animal models of Parkinson's

*Received 25 June 1998*
*Revised 6 November 1998*
*Accepted 25 November 1998*

*Correspondence to:* C Eng, Human Cancer Genetics Program, Ohio State University Comprehensive Cancer Center, 420 W 12th Avenue, 690C MRF, Columbus, OH 43210, USA

*Joint first authors.

disease, a disease in which dopaminergic neurons degenerate, treatment with GDNF has a protective effect (Lindsay, 1995; Moore et al, 1996). GDNF is, therefore, thought to play a key role in the control of vertebrate neuron survival and differentiation in both the central and peripheral nervous systems (Pichel et al, 1996).

Taking these data together, *GDNF* and *GFRα-1* appear to represent good targets for mutations which may play a pathogenetic role in the development of brain tumours. Additionally, the localization of *GFRα-1* to 10q26 (Gorodinsky et al, 1997; Eng et al, 1998), a region known to be somatically deleted at high frequency in malignant human brain tumours (Fults and Pedone, 1993), further supports its candidacy as a brain tumour gene.

### MATERIALS AND METHODS

### Patient samples

A panel of 36 sporadic brain tumours of various histologies and WHO grades (Table 1), from 36 unrelated German patients, was analysed. Tumour DNA was extracted from fresh frozen tissue and corresponding germline DNA from peripheral blood leucocytes, using standard protocols (Sambrook et al, 1989).

### Mutation analyses

Polymerase chain reaction (PCR) conditions and primers to amplify *GDNF* have been described (Dahia et al, 1997; Marsh et al, 1997). PCR of *GFRα-1* was carried out using 1 μM each of forward and reverse primer pairs (see below) in 1 × PCR buffer (Perkin-Elmer Corp.), 200 μM dNTP, 2.5 U *Taq* polymerase (Perkin-Elmer Corp.), TaqStart™ Antibodies (Clontech, San Francisco, CA, USA), and 100–200 ng of DNA template in a final volume of 50 μl. Reactions were subjected to 40 cycles of 94°C for 1 min, 58–62°C for 1 min and 72°C for 1 min followed by 10 min at 72°C. The PCR amplicons were then fractionated on 1% low melting point agarose (Bio-Rad Lab., Hercules, CA, USA) and visualized with UV transillumination after ethidium bromide staining. Before sequencing, these products were column purified (Wizard PCR Prep, Promega, Madison, WI, USA). Semi-automated sequencing was performed using either the forward or reverse primer and the ABI dye terminator cycle sequencing ready reaction kit as previously described (Liaw et al, 1997).

### GFRa-1 primers

Primers used to amplify *GFRα-1* for sequencing are:

Exon 1: RA-1F (5′-GTCGGACCTGAACCCCTAAAA-3′) (f)
RA-1R (5′-CCAAAAAGAAACTTCTTCCTTCC-3′)(r)
Exon 2: RAF22 (5′-GCAGACTTGCTCCTGTCGGC-3′) (f)
RAF7 (5′-CGCACGCTAAGGCAGTGCGT-3′) (f)
HRAR2N (5′-GGCTCTGGTACATGCTCCAGT-3′) (r)
HRAR2 (5′-CTGGTACATGCTCCAGTA-3′) (r)
Exon 3: RA-3F (5′-CAGCAAAAACCTGCTTGAAATA-3′) (f)
RAI-3F (5′-CAGCAAAAACCTGCTTGAAA-3′) (f)
RA-3R (5′-TGCCTCTTCATTATCATCATCCT-3′) (r)
RAI-3R (5′-TTCAAGCACACAAAGGCATC-3′) (r)
Exon 4: RAI-4F (5′-TGTGACCATGCCTGTCTTTC-3′) (f)
RAI-4R (5′-TCATTAATCACCAGCTGCCA-3′) (r)
Exon 5: RAI-5F (5′-CCCCACCCTTTTTCCTATTG-3′) (f)
RAI-5R (5′-CAGGCATGTCCTCAAGGATT-3′) (r)
Exon 6: GRI-1128-1F (5′-CTCAAGATAAATTGCCGA-GAAAAT-3′) (f)
RAI-6F (5′-GGCCATGGAAAAGTATCATCA-3′) (f)
GRI-1261-1R (5′-TACAGGCACAAGGTACAA-GAGGTA-3′) (r)
RAI-6R (5′-CTGGAGCTCGGAGAAGAAAA-3′) (r)
Exon 7: RAI-7F (5′-CGTTTGCTGCTTGACTTTGA-3′) (f)
RAI-7R (5′-GGAATCTGGACGCAGTTCTC-3′) (r)
Exon 8: RAI-8F (5′-TTTTTCTTGTCCCTCTCCAG-3′) (f)
RAR13 (5′-TCTATAAATGCACGAAGCCT-3′) (r)
Exon 9: GRI-1549-IF (5′-GCAGTGATGATAATGAAAC-CATTC-3′) (f)
GRA20R (5′-TTTTTCATGTCCATATTG-TATTTTT-3′) (r)

Table 1 WHO histologic classification of the 36 brain tumours

| | WHO grade | *n* |
|---|---|---|
| Glioblastoma multiforme | IV | 18 |
| Glioblastoma multiforme (recurrence) | IV | 3 |
| Oligoastrocytoma anaplastic | III | 1 |
| Astrocytoma anaplastic (recurrence) | III | 1 |
| Ependymoma | | 1 |
| Ependymoma anaplastic (recurrence) | III | 1 |
| Malignant peripheral nerve sheath tumour (MPNST) | | 1 |
| Meningioma atypical | | 2 |
| Meningioma anaplastic | III | 1 |
| Medulloblastoma | IV | 2 |
| Medulloblastoma (recurrence) | IV | 2 |
| Haemangiopericytoma | | 1 |
| Primitive neuroectodermal tumour (PNET, recurrence) | IV | 2 |

### Deletion analysis

DNA samples derived from brain tumours which were apparently homozygous for all *GFRα-1* polymorphisms underwent further analysis to determine if this represented hemizygous whole gene deletion. Corresponding germline DNA was examined for the presence of heterozygosity at the intragenic polymorphic sites that were apparently homozygous either by direct sequencing or differential restriction enzyme digestion. If the germline DNA was heterozygous for any one sequence variant, that tumour was defined as having loss of heterozygosity (LOH) of that marker, representing hemizygous gene deletion. If all intragenic polymorphisms were also homozygously present in the germline DNA, the result was not informative. In those cases, we performed a semi-quantitative duplex PCR using the tumour-derived DNA and primers for exon 2 of *GFRα-1* (RAF7, HRAR2) and those for the housekeeping gene beta-glucuronidase (GUSB) (Ivanchuk et al, 1997). The relative amount of the *GFRα-1* fragments versus that of *GUSB* were determined by visual inspection and densitometric scanning using ImageQuant software (Molecular Dynamics, Sunnyvale, CA, USA).

### RESULTS AND DISCUSSION

We analysed *GDNF* and *GFRα-1* for DNA sequence variants in a panel of 36 mainly high-grade human brain tumours by direct sequencing. No mutations were found in the coding region of *GDNF*. Analysing *GFRα-1*, we detected six distinct single nucleotide polymorphisms (Table 2) that have been previously

© Cancer Research Campaign 1999

**Table 2** *GFRα-1* germline frequencies of sequence variants in patients with brain tumours, HSCR disease and control DNA (Myers *et al.*, 1999)

| Amino acid change | Nucleotide | | Frequency | | |
|---|---|---|---|---|---|
| | | | Brain tumours | HSCR | Control |
| N/A | –106 | G | 0.93 | 0.96 | ND |
| | | A | 0.07 | 0.04 | ND |
| N/A | –78 | T | 0.67 | 0.69 | 0.68 |
| | | C | 0.33 | 0.31 | 0.32 |
| Y85N | 253 | T | 0.96 | 0.98 | 0.96 |
| | | A | 0.04 | 0.02 | 0.04 |
| N179N | 537 | T | 0.54 | 0.60 | 0.59 |
| | | C | 0.46 | 0.40 | 0.41 |
| N/A | IVS5+21 | G | 0.85 | 0.92 | 0.82 |
| | | A | 0.15 | 0.08 | 0.18 |
| T361A | 1081 | A | 0.91 | 0.93 | 0.88 |
| | | G | 0.09 | 0.07 | 0.12 |
| N/A | IVS8+28 | T | 0.97 | ND | ND |
| | | A | 0.03 | ND | ND |

N/A = not applicable, ND = not done.

found in the normal population (Myers et al, 1998). Two of these were in the 5′ untranslated region 106 bp (–106G>A) and 78 bp (–78T>C) upstream of the translational start site. One silent basepair substitution at codon 179 (c.537T>C) and a non-coding sequence polymorphism within intron 5 (IVS5+21G>A) were also observed. The remaining two polymorphisms were in exon 2 and exon 7 and resulted in amino acid substitutions (Y85N, 253T>A; T361A, 1081A>G). For codon 85, both the amino acids tyrosine and asparagine are neutral and polar. However, tyrosine is an aromatic, polar residue while the polymorphic asparagine is small and non-aromatic. Similarly, the substitution of threonine (neutral and polar) with alanine (neutral and hydrophobic) at codon 361 could also change the structure of GFRα-1. In those cases harbouring rare and/or apparently homozygous polymorphisms that lead to amino acid substitutions, it is conceivable that the stearic structure, and presumably, function, of GFRα-1 could be subtly altered such that the other co-receptors of RET (e.g. GFRα-2 or GFRα-3) may bind preferentially to GDNF and RET, thus leading to altered activation and/or specificity. We, therefore, examined the frequency of each of the *GFRα-1* single nucleotide polymorphisms in this series of patients with brain tumours and found them to be no different from those in normal controls or in non-cancer patients (Myers et al, 1999).

In addition to the six previously described single nucleotide polymorphisms, we also detected a homozygous non-coding sequence variant within intron 8, 28 basepairs downstream of the exon 8–intron 8 boundary (IVS8+28T>A) in the brain tumours of two unrelated patients (one glioblastoma multiforme, one anaplastic oligoastrocytoma). Interestingly, these two samples, together with 13 others, appeared to be homozygous at all six polymorphic sites. Further, the glioblastoma multiforme with the IVS8 sequence variant was apparently homozygous for the rare polymorphism at codon 361 (Table 2). Subsequent analyses of these 15 samples revealed somatic hemizygous deletion of *GFRα-1* in 11 of these 15 tumours (73% of 15; 31% of total), including both tumours with the intronic sequence variant.

In the present study, we did not find any obvious disease-associated somatic *GDNF* or *GFRα-1* intragenic mutations in DNA from human brain tumours from 36 individuals. We found seven single nucleotide polymorphisms within the genomic sequence of *GFRα-1* in this series, six of which have been previously noted (Myers et al, 1999). Of interest, we identified a novel intronic sequence variant in IVS8 in two patients. Our results indicate that intragenic mutations of *GDNF* and *GFRα-1* are not common aetiologic events in brain tumorigenesis. However, we did find that 31% of the 36 brain tumours had hemizygous *GFRα-1* deletion. This datum may support either of two postulates. First, it might well be possible that hemizygous deletion of *GFRα-1* is aetiologic in the pathogenesis of brain tumours. Second, the deletion of *GFRα-1* might be merely coincidental, an innocent bystander when large segments of chromosome 10q become deleted (Dalrymple et al, 1995; Simon et al, 1995; Albarosa et al, 1996). Putative tumour suppressor genes like *PTEN* or *DMBT* have been mapped in this region (Li et al, 1997; Mollenhauer et al, 1997; Steck et al, 1997; Duerr et al, 1999).

While it is obvious that 'high penetrance' mutations of *GDNF* and *GFRα-1* are not associated with brain tumorigenesis, it is becoming more and more evident that development of a cancer can result from an interplay of either a few 'high penetrance' mutations in key genes or from several, or many, sequence variants of unknown significance (Storey et al, 1998). In this respect, variant–variant interactions and/or variant–environment interactions may all be involved in predisposing to many common tumours. It is, therefore, intriguing that a few of these sequence variants in *GFRα-1* involve amino acids (Y85N, T361A) that are highly conserved among species (rat, chicken and human) [Genbank accession #U90541, #U59486, #U97144]. Further informatics-based and functional studies need to be performed to investigate whether these 'polymorphic' amino acid changes and seemingly neutral sequence variants have any impact on the function of this receptor.

### ACKNOWLEDGEMENTS

The Dana-Farber Cancer Institute Molecular Biology Core Facility is acknowledged for running the sequencing gels. OG is a recipient of a fellowship from the Deutsche Forschungsgesellschaft (DFG). CE is the Lawrence and Susan Marx Investigator in Human Cancer Genetics and a Barr Investigator.

### REFERENCES

Albarosa R, Colombo BM, Roz L, Magnani L, Pollo B, Cirenei N, Giani C, Conti AM, DiDonato S and Finocchiaro G (1996) Deletion mapping of gliomas suggest the presence of two small regions for candidate tumor-suppressor genes in a 17-cM interval on chromosome 10q. *Am J Hum Genet* **58**: 1260–1267

Baloh RH, Tansey MG, Golden JP, Creedon DJ, Heuckeroth RO, Keck CL, Zimonjic DB, Popescu NC, Johnson EM Jr. and Milbrandt J (1997) TmR2, a novel receptor that mediates neurturin and GDNF signaling through Ret. *Neuron* **18**: 793–802

Bogler O, Huang HJ, Kleihues P and Cavenee WK (1995) The p53 gene and its role in human brain tumors. *Glia* **15**: 308–327

Buj-Bello A, Adu J, Pinon LG, Horton A, Thompson J, Rosenthal A, Chinchetru M, Buchman VL and Davies AM (1997) Neurturin responsiveness requires a GPI-linked receptor and the Ret receptor tyrosine kinase. *Nature* **387**: 721–724

Dahia PL, Toledo SP, Mulligan LM, Maher ER, Grossman AB and Eng C (1997) Mutation analysis of glial cell line-derived neurotrophic factor (GDNF), a ligand for the RET/GDNF receptor alpha complex, in sporadic phaeochromocytomas. *Cancer Res*, **57**: 310–313

Dalrymple SJ, Herath JF, Ritland SR, Moertel CA and Jenkins RB (1995) Use of fluorescence in situ hybridization to detect loss of chromosome 10 in astrocytomas. *J Neurosurg* **83**: 316–323

Davies AM, Dixon JE, Fox GM, Ibanez CF, Jing S, Johnson E, Milbrandt J, Phillips H, Rosenthal A, Saarma M, Sanicola M, Treanor J and Vega QC (1997)

© Cancer Research Campaign 1999

Nomenclature of GPI-linked receptors for the GDNF ligand family. GFR(alpha) Nomenclature Committee [letter]. *Neuron* **19**: 485

Duerr EM, Rollbrocker B, Hayashi Y, Peters N, Meyer-Puttlitz B, Louis DN, Schramm J, Wiestler OD, Parsons R, Eng C and von Deimling A (1998) PTEN mutations in gliomas and glioneuronal tumors. *Oncogene* **16**: 2259–2264

Durbec P, Marcos-Gutierrez CV, Kilkenny C, Grigoriou M, Wartiowaara K, Suvanto P, Smith D, Ponder B, Costantini F, Saarma M, Sariola H and Pachnis V (1996) GDNF signalling through the Ret receptor tyrosine kinase [see comments]. *Nature* **381**: 789–793

Eng C, Myers SM, Kogon MD, Sanicola M, Hession C, Cate RL and Mulligan LM (1998) Genomic structure and chromosomal localization of the human GDNFR-alpha gene. *Oncogene* **16**: 597–601

Enokido Y, de Sauvage F, Hongo JA, Ninkina N, Rosenthal A, Buchman VL and Davies AM (1998) GFR alpha-4 and the tyrosine kinase Ret form a functional receptor complex for persephin [In Process Citation]. *Curr Biol* **8**: 1019–1022

Fults D and Pedone C (1993) Deletion mapping of the long arm of chromosome 10 in glioblastoma multiforme. *Genes Chromosomes Cancer* **7**: 173–177

Gorodinsky A, Zimonjic DB, Popescu NC and Milbrandt J (1997) Assignment of the GDNF family receptor alpha-1 (GFRA1) to human chromosome band 10q26 by in situ hybridization. *Cytogenet Cell Genet* **78**: 289–290

Hudson J, Granholm AC, Gerhardt GA, Henry MA, Hoffman A, Biddle P, Leela NS, Mackerlova L, Lile JD, Collins F and Hoffer BJ (1995) Glial cell line-derived neurotrophic factor augments midbrain dopaminergic circuits in vivo. *Brain Res Bull* **36**: 425–432

Ivanchuk SM, Eng C, Cavenee WK and Mulligan LM (1997) The expression of RET and its multiple splice forms in developing human kidney. *Oncogene* **14**: 1811–1818

Jing S, Wen D, Yu Y, Holst PL, Luo Y, Fang M, Tamir R, Antonio L, Hu Z, Cupples R, Louis JC, Hu S, Altrock BW and Fox GM (1996) GDNF-induced activation of the ret protein tyrosine kinase is mediated by GDNFR-alpha, a novel receptor for GDNF. *Cell* **85**: 1113–1124

Jing S, Yu Y, Fang M, Hu Z, Holst PL, Boone T, Delaney J, Schultz H, Zhou R and Fox GM (1997) GFRalpha-2 and GFRalpha-3 are two new receptors for ligands of the GDNF family. *J Biol Chem* **272**: 33111–33117

Klein RD, Sherman D, Ho WH, Stone D, Bennett GL, Moffat B, Vandlen R, Simmons L, Gu Q, Hongo JA, Devaux B, Poulsen K, Armanini M, Nozaki C, Asai N, Goddard A, Phillips H, Henderson CE, Takahashi M and Rosenthal A (1997) A GPI-linked protein that interacts with Ret to form a candidate neurturin receptor. *Nature* **387**: 717–721

Kotzbauer PT, Lampe PA, Heuckeroth RO, Golden JP, Creedon DJ, Johnson EM, Jr. and Milbrandt J (1996) Neurturin, a relative of glial-cell-line-derived neurotrophic factor. *Nature* **384**: 467–470

Li J, Yen C, Liaw D, Podsypanina K, Bose S, Wang SI, Puc J, Miliaresis C, Rodgers L, McCombie R, Bigner SH, Giovanella BC, Ittmann M, Tycko B, Hibshoosh H, Wigler MH and Parsons R (1997) PTEN, a putative protein tyrosine phosphatase gene mutated in human brain, breast, and prostate cancer [see comments]. *Science* **275**: 1943–1947

Liaw D, Marsh DJ, Li J, Dahia PL, Wang SI, Zheng Z, Bose S, Call KM, Tsou HC, Peacocke M, Eng C and Parsons R (1997) Germline mutations of the PTEN gene in Cowden disease, an inherited breast and thyroid cancer syndrome. *Nat Genet* **16**: 64–67

Lin LF, Doherty DH, Lile JD, Bektesh S and Collins F (1993) GDNF: a glial cell line-derived neurotrophic factor for midbrain dopaminergic neurons [see comments]. *Science* **260**: 1130–1132

Lindsay RM (1995) Neuron saving schemes [news; comment]. *Nature* **373**: 289–290

Marsh DJ, Zheng Z, Arnold A, Andrew SD, Learoyd D, Frilling A, Komminoth P, Neumann HP, Ponder BA, Rollins BJ, Shapiro GI, Robinson BG, Mulligan LM and Eng C (1997) Mutation analysis of glial cell line-derived neurotrophic factor, a ligand for an RET/coreceptor complex, in multiple endocrine neoplasia type 2 and sporadic neuroendocrine tumors. *J Clin Endocrinol Metab* **82**: 3025–3028

Milbrandt J, de Sauvage FJ, Fahrner TJ, Baloh RH, Leitner ML, Tansey MG, Lampe PA, Heuckeroth RO, Kotzbauer PT, Simburger KS, Golden JP, Davies JA, Vejsada R, Kato AC, Hynes M, Sherman D, Nishimura M, Wang LC, Vandlen R, Moffat B, Klein RD, Poulsen K, Gray C, Garces A, Henderson CE, Phillips HS and Johnson EM, Jr. (1998) Persephin, a novel neurotrophic factor related to GDNF and neurturin. *Neuron* **20**: 245–253

Mollenhauer J, Wiernann S, Scheurlen W, Korn B, Hayashi Y, Wilgenbus KK, von Deimling A and Poustka A (1997) DMBT1, a new member of the SRCR superfamily, on chromosome 10q25.3–26.1 is deleted in malignant brain tumours. *Nat Genet* **17**: 32–39

Moore MW, Klein RD, Farinas I, Sauer H, Armanini M, Phillips H, Reichardt LF, Ryan AM, Carver-Moore K and Rosenthal A (1996) Renal and neuronal abnormalities in mice lacking GDNF. *Nature* **382**: 76–79

Myers SM, Salomon R, Gössling A, Pelet A, Eng C, von Deimling A, Lyonnet S and Mulligan LM (1999) Absence of germline GFRalpha-1 mutations in Hirschsprung disease. *J Med Genet* (in press)

Naveilhan P, Baudet C, Mikaels A, Shen L, Westphal H and Ernfors P (1998) Expression and regulation of GFRalpha3, a glial cell line-derived neurotrophic factor family receptor. *Proc Natl Acad Sci USA* **95**: 1295–1300

Peters N, Wellenreuther R, Rollbrocker B, Hayashi Y, Meyer-Puttlitz B, Duerr EM, Lenartz D, Marsh DJ, Schramm J, Wiestler OD, Parsons R, Eng C and von Deimling A (1998) Analysis of the PTEN gene in human meningiomas. *Neuropathol Appl Neurobiol* **24**: 3–8

Pichel JG, Shen L, Sheng HZ, Granholm AC, Drago J, Grinberg A, Lee EJ, Huang SP, Saarma M, Hoffer BJ, Sariola H and Westphal H (1996) Defects in enteric innervation and kidney development in mice lacking GDNF. *Nature* **382**: 73–76

Sambrook J, Fritsch EF and Maiatis T (1989) Molecular Cloning: A laboratory Manual. Cold Spring Harbor Laboratories: Cold Spring Harbor

Sanchez MP, Silos-Santiago I, Frisen J, He B, Lira SA and Barbacid M (1996) Renal agenesis and the absence of enteric neurons in mice lacking GDNF. *Nature* **382**: 70–73

Sanicola M, Hession C, Worley D, Carmillo P, Ehrenfels C, Walus L, Robinson S, Jaworski G, Wei H, Tizard R, Whitty A, Pepinsky RB and Cate RL (1997) Glial cell line-derived neurotrophic factor-dependent RET activation can be mediated by two different cell-surface accessory proteins. *Proc Natl Acad Sci USA* **94**: 6238–6243

Schuchardt A, D'Agati V, Larsson-Blomberg L, Costantini F and Pachnis V (1994) Defects in the kidney and enteric nervous system of mice lacking the tyrosine kinase receptor Ret [see comments]. *Nature* **367**: 380–383

Simon M, von Deimling A, Larson JJ, Wellenreuther R, Kaskel P, Waha A, Warnick, RE, Tew JM Jr. and Menon AG (1995) Allelic losses on chromosomes 14, 10, and 1 in atypical and malignant meningiomas: a genetic model of meningioma progression. *Cancer Res* **55**: 4696–4701

Steck PA, Pershouse MA, Jasser SA, Yung WK, Lin H, Ligon AH, Langford LA, Baumgard ML, Hattier T, Davis T, Frye C, Hu R, Swedlund B, Teng DH and Tavtigian SV (1997) Identification of a candidate tumour suppressor gene, MMAC1, at chromosome 10q23.3 that is mutated in multiple advanced cancers. *Nat Genet* **15**: 356–362

Storey A, Thomas M, Kalita A, Harwood C, Gardiol D, Mantovani F, Breuer J, Leigh IM, Matlashewski G and Banks L (1998) Role of a p53 polymorphism in the development of human papillomavirus-associated cancer [see comments]. *Nature* **393**: 229–234

Stromberg I, Bjorklund L, Johansson M, Tomac A, Collins F, Olson L, Hoffer B and Humpel C (1993) Glial cell line-derived neurotrophic factor is expressed in the developing but not adult striatum and stimulates developing dopamine neurons in vivo. *Exp Neurol* **124**: 401–412

Takahashi M and Cooper GM (1987) ret transforming gene encodes a fusion protein homologous to tyrosine kinases. *Mol Cell Biol* **7**: 1378–1385

Thompson J, Doxakis E, Pinon LG, Strachan P, Buj-Bello A, Wyatt S, Buchman VL and Davies AM (1998) GFRalpha-4, a new GDNF family receptor. *Mol Cell Neurosci* **11**: 117–126

Tomac A, Lindqvist E, Lin LF, Ogren SO, Young D, Hoffer BJ and Olson L (1995) Protection and repair of the nigrostriatal dopaminergic system by GDNF in vivo [see comments]. *Nature* **373**: 335–339

Treanor JJ, Goodman L, de Sauvage F, Stone DM, Poulsen KT, Beck CD, Gray C, Armanini MP, Pollock RA, Hefti F, Phillips HS, Goddard A, Moore MW, Buj-Bello A, Davies AM, Asai N, Takahashi M, Vandlen R, Henderson CE and Rosenthal A (1996) Characterization of a multicomponent receptor for GDNF [see comments]. *Nature* **382**: 80–83

Trupp M, Arenas E, Fainzilber M, Nilsson AS, Sieber BA, Grigoriou M, Kilkenny C, Salazar-Grueso E, Pachnis V, Arumae U, Sariola H, Saarma M and Ibanez CF (1996) Functional receptor for GDNF encoded by the c-ret protooncogene [see comments]. *Nature* **381**: 785–788

Vega QC, Worby CA, Lechner MS, Dixon JE and Dressler GR (1996) Glial cell line-derived neurotrophic factor activates the receptor tyrosine kinase RET and promotes kidney morphogenesis. *Proc Natl Acad Sci USA* **93**: 10657–10661

von Deimling A, Louis DN and Wiestler OD (1995) Molecular pathways in the formation of gliomas. *Glia* **15**: 328–338

Wang SI, Puc J, Li J, Bruce JN, Caims P, Sidransky D and Parsons R (1997) Somatic mutations of PTEN in glioblastoma multiforme. *Cancer Res* **57**: 4183–4186

Weiner HL (1995) The role of growth factor receptors in central nervous system development and neoplasia. *Neurosurgery* **37**: 179–193; discussion 193–194

Worby CA, Vega QC, Chao HH, Seasholtz AF, Thompson RC and Dixon JE (1998) Identification and characterization of GFRalpha-3, a novel Co-receptor belonging to the glial cell line-derived neurotrophic receptor family. *J Biol Chem* **273**: 3502–3508

 *© Cancer Research Campaign 1999*
